# Anomaly Detection in Optical Coherence Tomography Angiography (OCTA) with a Vector-Quantized Variational Auto-Encoder (VQ-VAE)

**DOI:** 10.3390/bioengineering11070682

**Published:** 2024-07-05

**Authors:** Hana Jebril, Meltem Esengönül, Hrvoje Bogunović

**Affiliations:** 1 Lab for Ophthalmic Image Analysis, Department of Ophthalmology and Optometry, Medical University of Vienna, 1090 Vienna, Austria; hana.jebril@meduniwien.ac.at (H.J.); meltem.esengoenuel@meduniwien.ac.at (M.E.); 2 Christian Doppler Lab for Artificial Intelligence in Retina, Department of Ophthalmology and Optometry, Medical University of Vienna, 1090 Vienna, Austria

**Keywords:** OCTA, anomaly detection, VQ-VAE, epistemic uncertainty, deep learning, retina, ophthalmology

## Abstract

Optical coherence tomography angiography (OCTA) provides detailed information on retinal blood flow and perfusion. Abnormal retinal perfusion indicates possible ocular or systemic disease. We propose a deep learning-based anomaly detection model to identify such anomalies in OCTA. It utilizes two deep learning approaches. First, a representation learning with a Vector-Quantized Variational Auto-Encoder (VQ-VAE) followed by Auto-Regressive (AR) modeling. Second, it exploits epistemic uncertainty estimates from Bayesian U-Net employed to segment the vasculature on OCTA en face images. Evaluation on two large public datasets, DRAC and OCTA-500, demonstrates effective anomaly detection (an AUROC of 0.92 for the DRAC and an AUROC of 0.75 for the OCTA-500) and localization (a mean Dice score of 0.61 for the DRAC) on this challenging task. To our knowledge, this is the first work that addresses anomaly detection in OCTA.

## 1. Introduction

Based on the World Health Organization (WHO), at least 2.2 billion people have a near or distance vision impairment, and in approximately 1 billion of these, vision impairment could have been prevented or is yet to be addressed. Furthermore, about 4% of the global population suffers from severe vision impairments [1]. This translates to around 300 million individuals worldwide. The leading causes of these issues are various retinal diseases including Age-related Macular Degeneration (AMD), glaucoma, and Diabetic Retinopathy (DR). Aging with AMD as well as diabetes with DRP, severely affect the retina, resulting in world-wide vision loss increasing by 24% for severe vision loss over a decade [2]. This impairment significantly impacts individuals and the healthcare systems. These are not only prevalent but also progressive conditions that can lead to irreversible vision loss if left untreated. The effective management of these diseases requires not only medical intervention but also public health strategies to ensure early detection and access to appropriate treatments, thereby reducing the burden on healthcare systems worldwide [3].

In clinical diagnosis and treatment of retinal diseases, it is often essential to visualize the blood flow in the retinal vasculature, where the fundus fluorescein angiography (FA) imaging technique is commonly used [4]. This photographic imaging is acquired through a bandpass filter following the excitation of an extrinsic fluorophore by injecting the patient with fluorescein sodium in the bloodstream through a vein. The main issue of FA is the negative reactions in patients to the contrast agent, such as vomiting, or acute hypertension during and after the imaging process [5].

Modern multimodal retinal imaging techniques have emerged in the last decades, such as optical coherence tomography (OCT) [6] and OCT Angiography [7]. The output of these non-invasive imaging modality techniques is dense volumetric scans of high dimensionality and resolution. In particular, OCTA is a non-invasive imaging modality that is produced by processing the OCT data to provide microcirculatory imaging. It works by acquiring several B-scans at the same anatomical location repeatedly in short succession, followed by speckle change quantification between these B-scans [8]. Compared to FA, OCTA is non-invasive and offers greater precision in assessing the foveal avascular zone (FAZ) and areas of capillary non-perfusion [9]. From the clinical point of view, it provides a clearer visualization of the deep capillary plexus and choroid, which is needed to assess the capillary dropout and the flow voids in the retina [10].

Unlike the structural OCT, OCTA provides rich information on blood flow in retinal vasculature and choroid and can visualize ocular and systemic disease-related early changes. The extracted computational imaging biomarkers are commonly used to detect ocular diseases such as AMD and DR [7]. In parallel, these biomarkers allow for the discovery of non-ocular systemic diseases and predict their severity, such as cardiovascular disease (CVD) and coronary kidney disease (CKD) [11].

Retinal OCTA imaging offers several benefits that make it an ideal candidate for artificial intelligence (AI) applications. However, OCTA images often contain artifacts that can diminish these advantages [12]. These artifacts are caused by the scanner such as device calibration, patient-related factors such as eye blinking and lens opacity, or other similar issues that can cause a loss of information, resulting in motion or shadowing artifacts [13]. Despite this, numerous deep learning studies focus on OCTA, tackling tasks such as automated segmentation of non-perfusion areas, feature analysis of OCTA images (including vessel density), and retinal neovascularisation detection, segmentation, and quantification [14]. Moreover, OCTA images find utility in diagnosing and classifying diseases [15], with a primary focus on detecting retinal diseases such as diabetic retinopathy (DR) through disease classification methods like support vector machines (SVMs) [16], convolutional neural network (CNN) classifiers [17,18], or DenseNet [19], and age-related macular degeneration (AMD) detection via CNN networks [20]. In segmentation tasks, the predominant approaches involve segmenting the vasculature of the foveal avascular zone (FAZ) region, often employing models like U-Net [21] or ResNet [22]. In addition, several works focus on DR lesion segmentation using transfer learning [23] and U-Net with strong augmentation [24]. A recent approach jointly segments and classifies DR lesions by using a task-specific network (TSNet) with attention blocks [25]. These works only focus on specific diseases that require a dataset including each disease.

Automated diagnosis of retinal diseases with AI systems is considered challenging because some diseases such as CVD have several retinal microvascular abnormalities [26]. Furthermore, training AI algorithms to detect common retinal diseases requires a large-scale annotated dataset, while datasets of rare diseases are difficult to collect in sufficient size for training. In contrast, unsupervised deep learning requires a dataset of only healthy images that can be obtained fairly easily. Therefore, leveraging the information from normal data to detect abnormal behaviour using the anomaly detection paradigm is a widely studied problem [27]. One of the main advantages of the anomaly detection approach in the context of OCTA is that such models are not limited to a specific biomarker or disease, as not all imaging patterns in OCTA are interpretable and known.

Herein we present an unsupervised anomaly detection approach for OCTA images, based on two complementary deep learning models: A Vector-Quantized Variational Auto-Encoder (VQ-VAE) connected with Auto-Regressive (AR) modeling, and a Bayesian U-Net for blood vessel segmentation. Both models are trained using 2D full projection OCTA en face images of healthy subjects only. To the best of our knowledge, this is the first anomaly detection effort proposed for OCTA en face images.

### 1.1. Related Work

Identifying anomalies plays a vital role in medical imaging, as it enables the detection of irregular structures or patterns within medical images [28]. These anomalies often serve as indicators of underlying diseases or health-related issues. Thus, in the last decades, a wide range of anomaly detection algorithms have been proposed in the medical field for different types of medical data like magnetic resonance (MR) images [29,30] and X-rays [31]. There are different categories for deep learning anomaly detection, such as feature extraction and reconstruction-based approaches. In the feature extraction, relevant features are extracted from the input data, and anomalies are detected based on deviations from expected patterns in these features [32,33]. On the other hand, in the reconstruction-based approach, anomalies are detected by reconstructing input data and comparing the reconstructed data with the original input. Anomalies are identified based on large reconstruction errors [34,35].

In retinal imaging, several unsupervised anomaly detection studies have concentrated on color fundus and OCT images. In contrast, OCTA images are still quite a new imaging modality yet to be investigated. All previous anomaly detection methods focused on B scans as cross-sectional slices from structural OCT volumes. A popular OCT reconstruction-based anomaly detection model is the F-AnoGAN [34] approach, which introduces a method for unsupervised anomaly detection using Generative Adversarial Networks (GANs). F-AnoGAN leverages GANs to learn the underlying distribution of normal data in an unsupervised manner. The model learns to generate realistic samples from the normal data distribution, allowing it to identify anomalies as instances that deviate significantly from this learned distribution. In the testing phase, unseen data is given to the model along with a ground truth label to evaluate the anomaly detection performance, and this is done by mapping the latent space in each iteration and computing the loss.

Furthermore, Sparse-GAN [36] introduces a reconstruction-based anomaly detection approach in retinal OCT B-scan images by incorporating sparsity constraints into the GAN framework. During training, a GAN architecture is trained on normal retinal OCT images, with the generator enforced to produce sparse representations. This encourages the generator to generate images with localized anomalies, while the discriminator distinguishes between real and generated images. During testing, input images are fed into the trained generator, and anomaly scores are computed based on the difference between the input image and its generated counterpart. A threshold is applied to classify images as normal or anomalous. However, because of their vascular elongated and high-gradient structure, OCTA en face images are difficult to reconstruct in both the F-AnoGAN and Sparse-GAN approaches. In [37], epistemic uncertainty is used to exploit anomalies by training a Bayesian U-Net on healthy B-scans to segment their weak retinal layer structure. During testing, the Monte Carlo dropout sampling method is utilized to compute the epistemic uncertainty maps to be post-processed by the majority-ray-casting method to acquire the anomaly segmentation final result.

A feature extraction-based anomaly detection approach using OCT B-scans, proposed in [33], divides the problem into two main steps. The first step is the feature learning step that uses a deep denoising autoencoder (DDAE) to learn features representative of healthy OCT B-scans. The second phase is categorization by applying a one-class support vector machine (OC-SVM) to model the distribution of normal tissue features. The limitation of this approach is the need to determine a prior on the healthy amount of the volume. Similarly, another method presented in [38] utilizes anomaly detection on OCT scans by employing a Gaussian Mixture Model (GMM) to represent the global appearance of healthy OCT B-scan images, and subsequently identify diseased images as outliers. However, a notable constraint of this approach is its ability to solely detect anomalies at the scan level, lacking the capability of localizing the specific location of the anomaly within the image. None of these anomaly detection approaches incorporates the use of OCTA en face images.

Anomaly detection is a widely used technique across industries that highlights anomalies from natural images. One such widely cited approach is DRAEM (Discriminatively Trained Reconstruction Embedding for Surface Anomaly Detection) [39], designed specifically for identifying surface anomalies by integrating reconstruction and classification. Initially, DRAEM reconstructs input surface data to capture normal behaviour precisely. Subsequently, it employs a classifier trained on both normal and abnormal samples to discern regular variations from anomalies, presenting a dependable solution for surface anomaly detection. Nonetheless, a drawback of this method lies in its requirement for diverse anomaly samples during the discriminator training stage. Another method, CFA (Coupled-hypersphere-based Feature Adaptation) [40], introduces a transfer learning model tailored for anomaly localization through adapted features suited to the target dataset. CFA encompasses a trainable patch descriptor that extracts features from a normal dataset during training, storing them in a memory bank *C* independent of the target dataset size. During testing, each new patch undergoes comparison with patches stored in the memory bank using the nearest neighbour technique, facilitating the creation of an anomaly degree heatmap and the generation of a final anomaly score map. However, a limitation of this approach arises from the necessity for ground truth anomaly segmentation during the development phase.

### 1.2. Contribution

In this work, we investigate two distinct approaches to unsupervised anomaly detection in OCTA. First, we utilize VQ-VAE with AR models for unsupervised anomaly detection, requiring only en face images of healthy data. Second, we apply weakly labeled anomaly detection using a Bayesian U-Net to segment the vascular structure of OCTA en face images. The key contributions of this paper are as follows: (i) it marks a pioneering effort in anomaly detection on OCTA en face images, (ii) the proposed method demonstrates robustness to variations in OCTA scanner types and the field of view (FOV), and (iii) we provide a thorough evaluation of both scan-wise and pixel-wise anomaly detection on two large public datasets, offering comprehensive insights into the approach’s performance.

## 2. Methods

**Figure 1:**
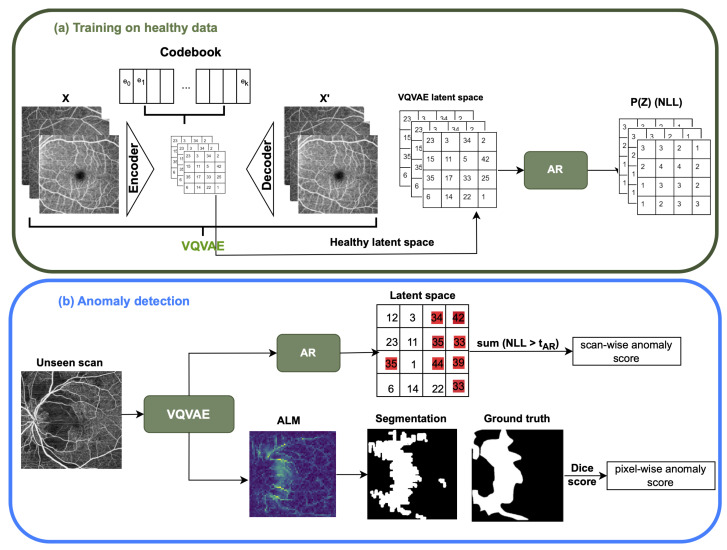
An outline of the VQ-VAE and AR method is provided. During the training phase, when the OCTA image *x* is provided as input to VQ-VAE, the output of the encoder is matched with the closest embedding vector from the VQ-VAE codebook to obtain the discrete latent space. Subsequently, the AR model learns the prior distribution to gauge the likelihood of samples. In the anomaly detection stage, VQ-VAE extracts the discrete spatial latent features. The AR model then assigns probabilities to each value in the latent space by using the negative log-likelihood (NLL) function, aggregating NLL of the latent space that is above a threshold $t_{ar}$ to derive the scan-wise score. Additionally, the VQ-VAE Alignment Loss Map (ALM) undergoes upscaling to generate a segmentation map for obtaining a pixel-wise score.

**Figure 2:**
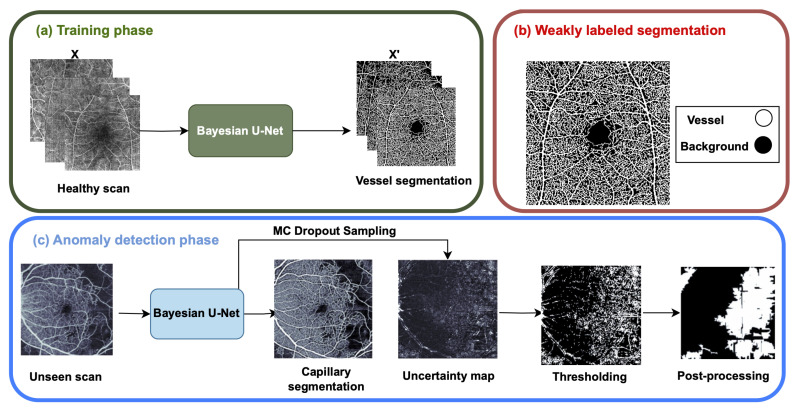
An overview of the epistemic uncertainty-based method. A Bayesian U-Net is trained on healthy OCTA en face projection images using vessel segmentation images on (**b**). During the anomaly detection phase, an unseen OCTA en face image is given. Monte Carlo dropout sampling is used to retrieve epistemic uncertainty maps, which are passed through thresholding and post-processing phases to obtain the final anomaly segmentation.

In this study, our focus lies on anomaly detection through two distinct methodologies. Initially, 2D OCTA en face projections are employed as they effectively capture the vascular features within the 3D volume. The first approach involves representational learning utilizing VQ-VAE alongside an AR model (Figure 1). Here, the VQ-VAE model is trained to generate healthy OCTA en face images, while the AR model assesses the VQ-VAE’s prior distribution to evaluate the probability of the latent space of the healthy scans. The second method entails employing a Bayesian U-Net Model (Figure 2). This model is trained to delineate the vascular structure of OCTA en face images in healthy subjects.

### 2.1. VQ-VAE and AR Model

This method comprises two main steps as illustrated in Figure 1. Specifically, the VQ-VAE [41] model is trained on healthy en face images, enabling encoding into a categorical latent space. Subsequently, the AR model [42] learns this latent distribution of healthy images, allowing the identification of an abnormal latent space associated with high prediction errors. Our method proficiently detects anomalies in retinal vascular perfusion based on the probability of the latent space pixel value; importantly, an anomalous pixel will exhibit a low probability of occurrence in standard data. Additionally, a segmentation result is obtained from the VQ-VAE Alignment Loss Map (ALM), indicating the VQ-VAE codebook loss, where anomalies will result in significant distances from codebook vectors. More detailed explanations of these models can be found in Section 2.1.1 and Section 2.1.2. This approach is inspired from [43] with an addition of using ALM for pixel-wise results.

#### 2.1.1. The Vector Quantized Variational AutoEncoder (VQ-VAE)

VQ-VAE compresses the input image *x* into discrete latent variables. The main idea of such an autoencoder is that both the encoder and decoder have a shared component called codebook. This codebook comprises *K* prototype vectors $e_{k},k\in 1,2\ldots K$. Hence, the encoder comprises input *x* into an E(x) matrix, and the codebook vectors are then employed to quantize the encoder matrix based on its distance by replacing it with the nearest prototype vector index in the codebook. The decoder input is the corresponding quantization output from Equation (1) to reconstruct the input image *x* to $x^{\prime }$ via a nonlinear function.

(1) $$\mathrm{Quantize}\left(E\left(x\right)\right)=e_{k},\mathrm{where}\;k=\underset{j}{arg min}∥E\left(x\right)-e_{j}∥$$

The VQ-VAE introduces two additional loss terms beyond the reconstruction loss of the VAE model. Firstly, the codebook alignment loss exclusively affects the codebook vectors to ensure their proximity to the encoder output. Secondly, the commitment loss prevents the encoder weights from frequently switching between code vectors. The following equation depicts the comprehensive loss function:

(2) $$L\left(x,D\left(e\right)\right)=||x-{D\left(e\right)||}_{2}^{2}+\underset{alignment\;loss}{\underset{︸}{||sg\left[E\left(x\right)\right]-{e||}_{2}^{2}}}+||sg\left[e\right]-{E\left(x\right)||}_{2}^{2}$$

where *e* is the quantized code for the input *x*. The functions *E* and *D* represent the encoder and the decoder, respectively. The operator sg is the stop gradient operation that prevents the gradient from flowing into its arguments.

Importantly, alignment loss is a highly robust and interpretable method for identifying anomalies. This is because, during training, the codebook vectors are trained to align with the encoder output closely. Consequently, during the testing phase, all of the anomalous regions are expected to be distinctly distant from the codebook vectors.

#### 2.1.2. The Auto-Regressive (AR) Model

AR is a generative probabilistic model utilized for high dimensional data $x=(x_{1},\ldots ,x_{n})$ to model the joint probability distribution of one variable at a time, which only depends on the previous variables, as illustrated in Equation (3).

(3) $$p\left(x\right)=p\left(x_{1},\ldots ,x_{n}\right)=\prod_{i=1}^{n}p\left(x_{i}|x_{1},\ldots ,x_{i-1}\right)$$

In our work, the AR model PixelSNAIL [42] learned the prior distribution of the VQ-VAE to estimate the probability distribution of healthy data by using the NLL probability function. Consequently, the anomalous scan can be identified by summing the NLL of the latent space that is above a threshold that is assigned empirically.

#### 2.1.3. Implementation Details

Our VQ-VAE model consists of 5 convolutional blocks, each composed of 4 residual blocks. The VQ-VAE codebook contains 128 keys with 256 dimensions, and the latent space array size is set to 60×60. During training, we utilize the Adam optimizer with a learning rate of $1\times 10^{-4}$ and incorporate dropout with a probability of 0.1. Training extends over 200 epochs with a batch size of 4, using L1 loss as the reconstruction loss function. In contrast, the AR PixelSNAIL model comprises 4 convolutional blocks, each incorporating a self-attention module and 4 residual blocks. This model is trained using the Adam optimizer with a learning rate of $5\times 10^{-4}$, over 300 epochs, and employs cross-entropy loss. To augment the training dataset, we employ techniques such as horizontal flipping, random resize crop, rotation, and Gaussian blur, which help increase the diversity of input images. Two important hyperparameters, $t_{al}$ and $t_{ar}$, are crucial in our anomaly detection process. $t_{al}$ is fixed at 0.05 and serves as a threshold for identifying anomalous pixels based on their alignment loss value from the VQ-VAE model. On the other hand, $t_{ar}$ is set at 7, corresponding to the 95th percentile, and determines the threshold for detecting anomalies within the scan latent space values at a scan level. Both thresholds are determined through analysis of the validation dataset to optimize anomaly detection performance.

### 2.2. The Epistemic Uncertainty of Bayesian U-Net Model

This algorithm comprises three main phases, illustrated in Figure 2. Firstly, a Bayesian U-Net model is trained on healthy OCTA en face projections to segment the OCTA vascular structure, utilizing weak labels instead of manual annotations, as shown in Figure 2b. Subsequently, the U-Net model employs the Monte Carlo dropout approach to obtain a map of pixel-level epistemic uncertainty estimates. This map undergoes post-processing to generate the final anomaly segmentation map. This approach was inspired by the work of [37], which focused on weak-label anomaly detection on structural OCT B-scans.

Section 2.2.1 provides details on training the model with weak labels, and Section 2.2.2 describes the epistemic uncertainty estimates for anomaly detection.

#### 2.2.1. Bayesian U-Net Training

In this study, we employed the U-Net model, widely recognized for its effectiveness in segmentation tasks. Let $X\in R^{a\times b}$ represent the set of healthy en face images, each with a pixel size of a×b, and let $y\in Y^{a\times b}$ correspond to the set of weak labels, denoting target labels for vessels and background, where Y=0,1.

**Figure 3:**
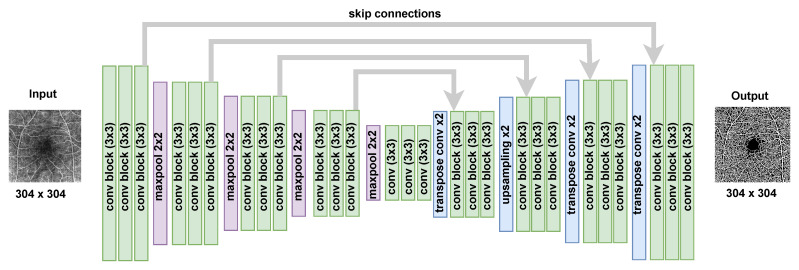
U-Net architecture for retinal vessel segmentation on OCTA en face images. Each convolutional block has the following structure: 3 × 3 convolution + batch-normalization + ReLU + Dropout.

The architecture of our U-Net instance (mentioned in [44]) is illustrated in Figure 3, featuring five levels of depth for both the encoder and decoder sections. Each convolutional block comprises a 3×3 convolution layer, followed by batch normalization and a Rectified Linear Unit (ReLU). Subsequently, dropout is applied after each convolutional block. The encoder downsampling employs a 2×2 max-pooling layer, while the decoder upsampling involves transpose convolution.

#### 2.2.2. Epistemic Uncertainty Map

During the test phase, applying dropout to obtain Monte Carlo samples and retrieve the dropout rate is a method for deriving the epistemic uncertainty in Bayesian models. Given an unseen en face image *x*, the epistemic uncertainty is computed by retrieving *n* predictions $y^{i},i=1,\ldots ,n$. Then, for each class *k* in *Y*, the pixel-wise variance $\sigma^{2}$ is computed.

(4) $$\sigma_{k}^{2}\left(P\right)=\frac{1}{n}\sum_{i}^{n}{\left(y_{k}^{i}\left(P\right)-\mu_{k}^{\;\;}\left(P\right)\right)}^{2}$$

where *P* is the pixel coordinates, and $\mu_{k}^{\;\;}$ is the mean of *n* predictions for each class *k*. Finally, the epistemic uncertainty map *u* is attained over all classes *K* by

(5) $$u\left(P\right)=\frac{1}{K}\sum_{k}^{K}\sigma_{k}^{2}\left(P\right)$$

#### 2.2.3. Implementation Details

For the training of the U-Net model in vessel segmentation, we employed the Adam optimizer with a learning rate of $5\times 10^{-4}$. The training process involved 200 epochs with a batch size of 4, and each convolutional block in the network utilized 64 channels, which is shown in Figure 3. The loss function, defined in Equation (6), incorporated weighted cross-entropy (CE) and Dice (DSC) losses between the input image *X* and the U-Net model’s output $\hat{X}$, as depicted in Figure 2a. The hyperparameter β was specifically set to 0.6, as recommended in  [44].

(6) $$L\left(X,\hat{X}\right)=\mathrm{CE}\left(X,\hat{X}\right)+\beta \mathrm{DSC}\left(X,\hat{X}\right)$$

To generate pixel-wise vessel segmentation labels for the DRAC dataset using the weakly labeled Bayesian U-Net model, we first trained a U2-Net model on the OCTA-500 dataset [45]. For this 180 6×6 mm${\;\;}^{2}$ en face images were used for training, resized to 320×320 and randomly cropped to 288×288 for input to the vessel segmentation model. The training employed the Adam optimizer with a learning rate of 0.001, combined cross-entropy and Dice loss functions, and utilized a batch size of 8 over 2000 training epochs. Additionally, in the anomaly detection method involving the Bayesian U-Net model, another crucial hyperparameter $t_{un}$ serves as a threshold to identify anomalous pixels based on their uncertainty map values. Following validation dataset analysis, $t_{un}$ was set to 0.

### 2.3. OCTA En Face Image Preprocessing

In our study, we preprocess the DRAC dataset OCTA en face images by downscaling them, while the OCTA-500 dataset OCTA en face images are upscaled, ensuring both datasets are standardized to a size of 512×512 pixels. Subsequently, we perform a center crop to adjust the images to a final size of 480×480 pixels, which helps eliminate artifacts typically found at the borders of en face projections. Additionally, we enhance the vasculature structure within the OCTA en face images using an approach based on the Hessian matrix and eigenvalues, as described in [46].

For better understanding, let $f:R^{N}\to R$ be the intensity values of the input OCTA en face image of *N* variables. The Hessian matrix, denoted by *H*, is given by

(7) $$H_{ij}=\frac{\partial^{2}f}{\partial x\partial y}$$

where *x* and *y* are the dimensions of the OCTA en face image.

To find the eigenvalues of the Hessian matrix, we solve the characteristic equation:

(8) det(H−λI)=0

where λ represents the eigenvalues, and *I* is the identity matrix. Eigenvalues provide crucial information about the behaviour of the function around critical points.

### 2.4. OCTA En Face Image Segmentation Post-Processing

In the first approach utilizing the VQ-VAE and AR model, the alignment loss map is scaled up to match the size of the original image. Conversely, in the second approach with the Bayesian U-Net, the uncertainty map is already generated at the original image size. To identify anomaly segmentation areas in both methods, we begin by thresholding the final map with a threshold $t_{al}$ for the VQ-VAE alignment loss map or $t_{un}$ for the uncertainty map. These thresholds are determined empirically based on the validation dataset to create a binary image. Subsequently, we apply morphological operations such as opening and dilation to enhance the segmentation map. Finally, small connected areas smaller than a specified area *s* are removed to produce the refined final segmentation map *B*.

## 3. Experimental Setup

### 3.1. Datasets

For method development and evaluation, we utilized two large publicly available OCTA datasets. We developed and internally evaluated our methods using the data from the DRAC [47] challenge and further externally evaluated them on an independent OCTA-500 [48] dataset.

The DRAC dataset comprises 1103 ultra-wide OCTA (UW-OCTA) en face images, each with a resolution of 1024×1024 pixels. This dataset is divided by the challenge organizers into a training set consisting of 611 images and a test set comprising 386 images. These training/test divisions adhere to the DR grading standards based on the DR severity scale, which ranges from non-DR and non-proliferative diabetic retinopathy (NPDR) to Proliferative Diabetic Retinopathy (PDR). Importantly, the dataset provides pixel-level annotations for several lesions: intraretinal microvascular abnormalities, non-perfusion areas, and neovascularization. These pixel-level annotations of pathological anomalies will allow us to evaluate our algorithm at the pixel level.

The OCTA-500 dataset is among the most comprehensive OCT/OCTA datasets available. It encompasses 500 subjects and is divided into two categories based on the acquired field of view (FOV) size: OCTA_6mm and OCTA_3mm. Moreover, each category contains not only 3D OCT/OCTA volumes but also six different en face projection maps derived from various retinal layers, as well as text labels with sex, age, and disease class. The OCTA en face projection map between the Internal Limiting Membrane (ILM) to the Outer Plexiform Layer (OPL) is used in this work along with the disease label from the text labels. In detail, the OCTA_6mm group consists of 300 subjects, with a mean age of 49.18±17.28. In this group, the proportion of subjects with a retinal disease is 69.79%. These diseases include AMD, DR, Choroidal Neovascularization (CNV), Central Serous Chorioretinopathy (CSC), Retinal Vein Occlusion (RVO), and others. On the other hand, the OCTA_3mm category comprises 200 subjects, primarily from a healthy population, with a disease proportion of only 20%. The diseases observed in this group are AMD, DR, and CNV, with the mean age of subjects included in the 3 mm group being 33.12±16.17. The dataset enables the detection of all diseases collectively as anomalies at the scan level.

In this study, we implemented our methods using only the DRAC training dataset because it has the ground truth labels, which is not the case for the OCTA-500 dataset. First, we selected the best quality images from the training dataset of 611 by removing the images with artifacts, based on the quality classification labels that are provided by the DRAC challenge, resulting in a total of 506 images. Then, we partitioned the dataset of 506 images into training, validation, and testing subsets. Specifically, healthy scans were distributed among these subsets as follows: 80% for training, 10% for validation, and 10% for testing. Diseased scans were equally divided between the validation and testing subsets, with each receiving 50%. Consequently, the training subset comprised 212 healthy scans, the testing subset included 148 scans, and the validation subset contained 146 scans. During training, a batch size of 4 was utilized. In addition, the OCTA-500 dataset comprising 500 scans is utilized as an additional testing dataset.

For the pixel-wise segmentation evaluation, we combined the pixel-level annotations of intraretinal microvascular abnormalities, non-perfusion areas, and neovascularization into a single image. Additionally, we excluded images with acquisition artifacts such as shadowing artifacts, as identified through manual inspection. We also removed images with very small anomalies or anomalies on the image borders, since cropping during reprocessing would eliminate these anomalies, ensuring the accuracy of our results. Consequently, we curated a validation dataset of 10 images and a test set of 31 images. It is important to note that these validation and test datasets were used exclusively during the pixel-wise segmentation evaluation phase.

### 3.2. The Anomaly Detection Evaluation Procedure

Our work employs two primary statistical analysis criteria for the anomaly detection evaluation:

- **Scan-wise anomaly score:** This score is associated with the probability that a given scan contains an anomaly. During model testing, abnormal regions in the input scan correspond to latent space NLL probability values in the AR model or uncertainty values for the Bayesian U-Net. The scan-wise score is the sum of threshold pixel values above a threshold $t_{ar}$ for the VQ-VAE with the AR model and above a threshold $t_{un}$ for the uncertainty map. Performance is evaluated using the area under the receiver operating characteristic curve (AUROC) score, along with the Average Precision (AP) score, which computes the average precision across all recall levels where both of these scores are computed a continuous values like our anomaly score. In addition, F1 score is computed based on selecting an operating point using the Youden Index to convert continuous score to a binary score.
- **Pixel-wise anomaly score:** The pixel-wise score quantifies the probability that a given pixel belongs to an anomalous region. In both approaches, pixel values that are above the $t_{al}$ or $t_{un}$ threshold in VQ-VAE ALM and Uncertainty Map, respectively, will be highlighted as anomaly pixels in the segmentation results. Evaluation is performed using the Dice similarity metric, which calculates the size of the intersection of two areas divided by the average size of the individual areas. Additionally, Intersection over Union (IOU), sensitivity (True Positive Rate), and specificity (True Negative Rate) are used for the evaluation.

## 4. Results

**Figure 4:**
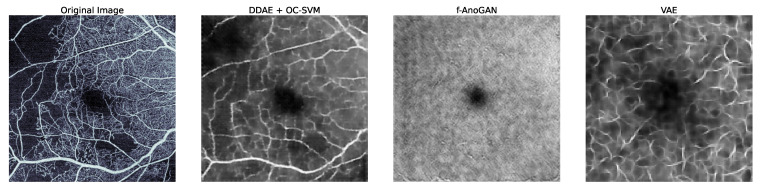
Examples of reconstructing normal variants of input scans for the baseline methods: the *DDAE with OC-SVM* approach reconstructed a denoised image; however, while keeping the abnormal areas, *F-AnoGAN* and *VAE* models produced poor reconstructions without recovering vascular structure.

For our anomaly detection approaches comparison, several baseline deep learning models were trained and evaluated, including *F-AnoGAN* [34], *DDAE with OC-SVM* [33], and *VAE* [49]. Figure 4 shows the reconstruction output for the baseline methods. Regarding the *F-AnoGAN* model, it was unable to learn and synthesize the vasculature, and the quality of the reconstruction was poor. Furthermore, the *DDAE with OC-SVM* approach could only reconstruct the input noised images into cleaned images while preserving the abnormal areas, as shown in Figure 4.

### 4.1. Scan-Wise Anomaly Detection Results

For the scan-wise anomaly score comparison, we utilized CFA [40] and DRAEM [39] approaches as baseline comparison algorithms. Notably, a minor modification was implemented in the CFA method to enhance the AUROC score. Specifically, instead of considering the maximum value in the heat map, we opted for the summation of the heat map values as the scan-wise score.

**Figure 5:**
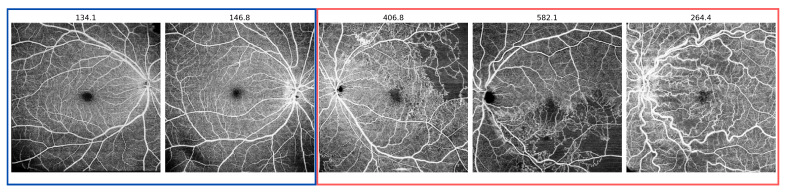
Examples of scans from the DRAC test set with the corresponding scan-wise anomaly score of the VQ-VAE with the AR model. Healthy images are denoted in blue, while diseased images are marked in red.

Regarding the qualitative results, we observed that the VQ-VAE with the AR model excelled in distinguishing between diseased and healthy images, surpassing the compared approaches. One significant observation was the model’s proficiency in identifying images with tortuous vessels as unhealthy scans, as illustrated in the final example in Figure 5.

**Figure 6:**
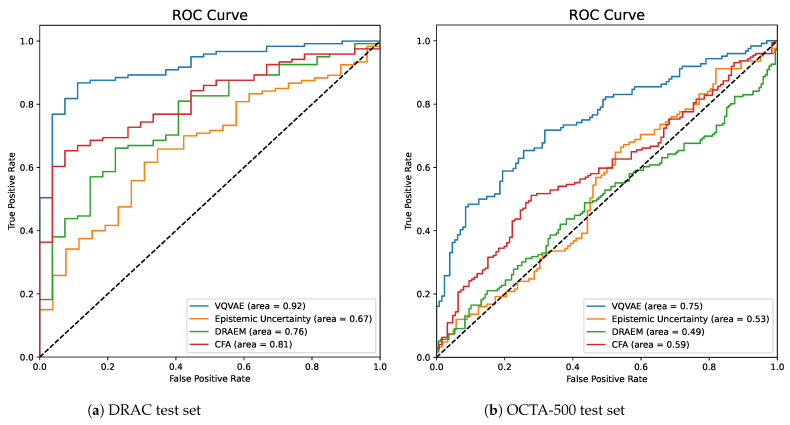
Results of scan-wise anomaly detection in the form of receiver-operating characteristic (ROC) curves. The dashed line denotes random performance.

For the quantitative results of the DRAC testing subset, Table 1 presents its classification results, comparing CFA [40], DRAEM [39], epistemic uncertainty-based Bayesian U-Net, and VQ-VAE with the AR models. This comparison indicates that the VQ-VAE with the AR model outperforms other models with an AUROC of 0.92, an F1 score of 0.92, and an AP of 0.98. Additionally, Figure 6 illustrates the ROC curve results and how the VQ-VAE with the AR approach outperforms other approaches. For further clarification, Figure 5 depicts the scan-wise score for various examples of healthy and diseased en face images from the DRAC independent test set. It vividly demonstrates how the scan-wise anomaly score is less than 150 for a healthy scan, whereas it exceeds 200 for a diseased scan.

**Figure 7:**
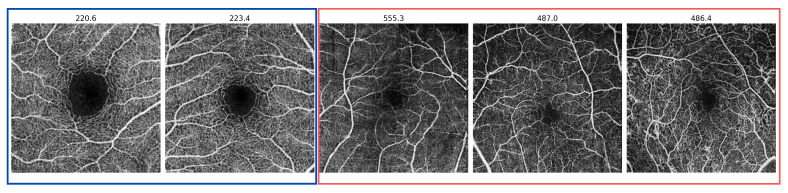
Examples of scan-wise anomaly score of the VQ-VAE with the AR model in the OCTA-500 dataset. Healthy images are indicated in blue, while diseased images are distinguished in red.

The OCTA-500 quantitative results are also presented in the same Table 1. From these results, it is evident that only the VQ-VAE with the AR approach was able to classify the OCTA-500 dataset with an AUROC of 0.75, an F1 score of 0.71, and an AP of 0.77. For more detailed insights, Figure 7 exhibits the scan-wise score for various examples of healthy and unhealthy en face images from the OCTA-500 dataset. It clearly illustrates how the scan-wise anomaly score is approximately 100 for a healthy scan, while in diseased scans it exceeds a value of 300.

### 4.2. Pixel-Level Anomaly Segmentation Results

The pixel-wise score results necessitated manually annotated images for quantifying scores, which are only available for diabetic disease within the DRAC dataset. Our approach is assessed on a subset of the DRAC dataset in the segmentation task for the pixel-wise score by calculating the DICE, IoU, Sensitivity, and Specificity scores, as detailed in the dataset in Section 3.1.

**Figure 8:**
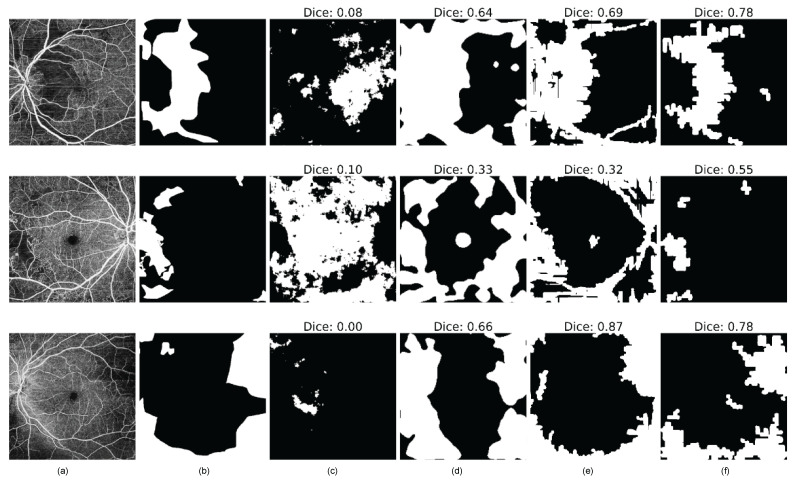
Results of pixel-wise anomaly segmentation on the DRAC testing subset. (**a**) Original image, (**b**) Groundtruth, (**c**) DRAEM [39], (**d**) CFA [40], (**e**) Bayesian U-Net, and (**f**) VQ-VAE ALM.

Qualitatively, both the VQ-VAE with AR and the Bayesian U-Net effectively pinpoint the location of anomalies within the images, as illustrated in Figure 8, when compared to the baseline methods. For clarity, the DICE score is computed between the segmentation results and the ground truth image. The findings underscore that the VQ-VAE ALM and the epistemic uncertainty approaches successfully segment anomalous regions.

For the quantitative results, Table 2 presents the assessment of pixel-wise anomaly scores among the tested models. The final score is derived from the average and standard deviation of the test samples. Both the epistemic uncertainty approach and the VQ-VAE ALM yield to the highest pixel-wise scores, with statistically significant differences, as indicated by *p*-values less than 0.001. Moreover, the VQ-VAE ALM excels in segmenting anomalous regions with greater specificity. In contrast, although the epistemic uncertainty approach achieves higher Dice, IoU, and sensitivity scores, the disparities between our two approaches were not statistically significant.

## 5. Discussion

In this work, we present an anomaly detection framework for OCTA en face projection 2D images. The framework aims to identify and localize anomalous regions in retinal perfusion. Our approach operates on two complementary fronts: first, employing VQ-VAE unsupervised anomaly detection, which relies solely on images of healthy cases. The second approach utilizes epistemic uncertainty-based Bayesian U-Net, leveraging the vascular structure of OCTA en face images, requiring manual annotations (weakly labeled anomaly detection). Recent research indicates that this is the first anomaly detection work specifically tailored to OCTA en face images.

Furthermore, the results of this work are categorized into classification and segmentation sections. In terms of classification, the VQ-VAE with the AR model approach outperforms baseline methods such as CFA, DRAEM, and the method based on uncertainty estimates. Notably, the trained VQ-VAE + AR method was shown to be successful when applied to an independent dataset (OCTA-500), without any fine-tuning. Moreover, the model can detect various anomalies, including retinal vessel tortuosity, which is a non-smooth appearance of the vessel course, as shown in Figure 5 (second example from the right). In the segmentation task, both the VQ-VAE ALM and the method based on uncertainty estimates outperform other methods, with higher specificity achieved by the VQ-VAE ALM, indicating the model’s ability to accurately highlight diseased areas. Compared to the baseline methods, our approach achieved significantly better results in terms of scan-wise and pixel-wise scores.

Compared to previous methods, such as DDAE with OC-SVM, F-AnoGAN, and VAE, our approach was more successful in identifying anomalies in OCTA en face images as shown in Figure 4. This is due to the greater complexity and detailed vascular structures in OCTA images compared to OCT B-scans. Additionally, our methodology outperforms the CFA and DRAEM industrial approaches, as the controlled nature of industrial settings makes it easier to detect anomalies, whereas highlighting anomalies in OCTA en face images is considerably more challenging.

For the segmentation task, we took advantage of applying the Hessian filter during evaluation, which enhances the vascular curvilinear structure of the OCTA. Additionally, using VQ-VAE ALM achieved more robust results than the VQ-VAE + AR model (Table 2). Our empirical observations showed that both the Hessian filter and VQ-VAE ALM only improved the segmentation results while worsening the classification results.

The primary advantages of using the VQ-VAE method include its ability to create a spatial and categorical latent space, which is utilized to identify abnormal pixels with the AR model. Additionally, this method requires only a dataset of healthy images, without the need for any annotations. However, the spatial resolution of the latent space is small relative to the original image size, making the model less effective at identifying tiny anomaly areas. In contrast, the epistemic uncertainty-based Bayesian U-Net excels by relying on traditional segmentation algorithms, which perform accurately in well-defined environments, such as within a healthy population. Nonetheless, this method still requires annotated labels, and obtaining manual annotatations of OCTA scans is costly, subjective, and time-consuming.

**Figure 9:**
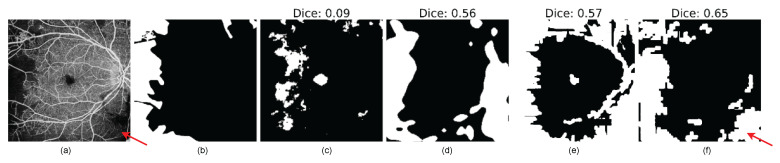
Example of OCTA artifacts being detected: in the input OCTA image, artifacts are observed in the bottom right corner, as indicated by the red arrow. These artifacts are represented as anomalous regions in the segmentation output. (**a**) Original image, (**b**) Groundtruth, (**c**) DRAEM [39], (**d**) CFA [40], (**e**) Bayesian U-Net, and (**f**) VQ-VAE ALM.

We identified certain limitations that require further development. The primary limitation is the model’s tendency to segment artifacts in OCTA images as anomalous areas, as shown in Figure 9, where the lower right corner of the image is misclassified as an anomaly. Another limitation is the lack of comprehensive pixel-level ground truth labels. For the OCTA-500 dataset, no pixel-level labels of diseased areas are available for the diseased cases. Moreover, only the regions affected by DR are provided by the DRAC dataset. These limitations call for the implementation of acquisition artifacts removal methods and emphasize the need for more comprehensively labeled datasets that distinguish between imaging artifacts and disease-related morphological changes.

In the future, we plan to implement our approach using 3D OCTA images to better capture the 3D vasculature, which is lost when projecting onto a single 2D en face image. This can be effectively achieved with our VQ-VAE method using AR. However, the Bayesian U-Net method will require 3D labels, which are difficult to manually annotate. Nonetheless, it may be feasible with a synthetic OCTA dataset, leveraging recent advancements in synthetic OCTA for detailed retinal vessel segmentation without human annotations [50].

In conclusion, OCTA en face images play a crucial role in the early detection and monitoring of ocular diseases as well as systemic diseases such as heart and kidney diseases. We have proposed a robust deep learning approach that can identify anomalies in OCTA projection maps. Such methods have the potential to assist in disease detection and patient management and support novel biomarker discovery in OCTA modality.

## Figures and Tables

**Table 1:** Results of scan-wise anomaly detection performance.

	DRAC			OCTA-500		
**Method**	**AUROC**	**F1**	**AP**	**AUROC**	**F1**	**AP**
CFA [40]	0.81	0.79	0.95	0.59	0.59	0.69
DRAEM [39]	0.76	0.77	0.93	0.49	0.26	0.55
Bayesian U-Net	0.67	0.75	0.90	0.53	0.6	0.58
VQ-VAE + AR	**0.92**	**0.92**	**0.98**	**0.75**	**0.71**	**0.77**

**Table 2:** Results of pixel-wise anomaly detection performance between CFA, DRAEM, Bayesian U-Net, and VQ-VAE ALM. ${\;\;}^{★★★}$ statistically significant with *p*-value <0.001.

Method	Dice	IoU	Sensitivity	Specificity
CFA [40]	$0.52\pm 0.17\;{\;\;}^{★★★}$	$0.37\pm 0.16\;{\;\;}^{★★★}$	$0.80\pm 0.08\;{\;\;}^{★★★}$	$0.67\pm 0.08\;{\;\;}^{★★★}$
DRAEM [39]	$0.11\pm 0.06\;{\;\;}^{★★★}$	$0.06\pm 0.03\;{\;\;}^{★★★}$	$0.16\pm 0.12\;{\;\;}^{★★★}$	$0.60\pm 0.23\;{\;\;}^{★★★}$
Bayesian U-Net	**0.61 ± 0.16**	**0.46 ± 0.17**	**0.80 ± 0.13**	0.80 ± 0.08
VQ-VAE ALM	0.60 ± 0.17	0.45 ± 0.17	0.57 ± 0.19	**0.91 ± 0.06**

## Data Availability

All the imaging datasets used in this paper are publicly available. The DRAC dataset is available from https://zenodo.org/records/10280359, accessed on 20 August 2022. The OCTA500 dataset is available from https://ieee-dataport.org/open-access/octa-500, accessed on 20 July 2021. The source code for both approaches is available upon reasonable request.
